# Chicago Medical Society

**Published:** 1887-02

**Authors:** 


### CHICAGO MEDICAL SOCIETY.

*Stated Meeting, December 20th, 1886.*—THE PRESIDENT, EDMUND J. DOERING, M.D., in the chair.

PROFESSOR HOSMER A. JOHNSON read a paper on PSEUDO-MEMBRANOUS BRONCHITIS, which appeared in the January number of the JOURNAL AND EXAMINER.

The record of statistics shows that a large majority are of a partial character, involving but a limited portion of the bronchial surface, and these, though obstinate and apparently difficult to relieve, usually recover. It is stated, also, that there is more than an ordinary tendency to involve ultimately the fibrous tissue of the lung and bring on that form of phthisis.

In regard to the treatment, he had nothing specially new to offer. He found that the chronic form was most benefited by ordinary anodyne expectorants combined with alteratives, especially of the mercurial class.

PROFESSOR ROBERT H. BABCOCK said the paper is one of the most interesting he had listened to. It was needless for him to say, since such a man as Doctor Johnson has seen but one case, and such a man as Doctor Bowditch has never seen a case, that he had never seen one. In regard to the etiology, he would merely state that R. Douglass Powell says this form of bronchitis may be observed in all ages from childhood to old age. He differs from the author of the paper in saying that it is more frequent in females than in males. He mentions the probability that there is a hyperinotic condition of the blood. The line of treatment pursued by Doctor Johnson is interesting with regard to this point; after giving iodide of potassium and the mercurial salt in pretty full doses the character of the expectoration changed, becoming mucous and frothy, and it was not until these remedies had been given up and syrup of iron substituted that the plastic character of the bronchitis reasserted itself. May it not be that the exhibition of the potassium iodide and the mercurial salt did overcome to a certain extent the hyperinosis which may have existed in this man's case?

PROFESSOR S. D. JACOBSON said it had been his good fortune to see, besides this instance of the chronic form, which he saw through the courtesy of Dr. Johnson, one case which represented the acute form of this disease. It was a friend of his, a man of about 47 years of age, who was accustomed to take great quantities of alcoholic stimulants. In the winter of 1871 he came to him and complained that for several days he had had some trouble with his pharynx, and he had expectorated a large amount of mucus. He inspected his pharynx and found a condition such as might be expected from a man who was out of doors a great deal, who was an inveterate smoker and a very convivial man. There were no symptoms of diphtheria; it was a case of pharyngitis or tonsillitis. Under the usual treatment it improved in a couple of days. About a week after he sent for Doctor Jacobson, and he found him with some fever and a very distressing cough. He expectorated great masses of mucus, which were recognized as casts of bronchial tubes of the first and second order. At that time he had never heard of such a case. The next day he found him in great distress, and after a long and tiresome cough he brought up numerous quantities of matter like the first casts observed, which were found to be of the third and fourth order of bronchial casts, and after that he felt relieved, but died on the third day afterward. He then saw a report in the

*Scandinavian Medical Archive*[1] describing a case exactly similar to this, except that the man was 34 years of age. On post-mortem it was found by incising the chest that it contained air over both lungs, and the bronchial tubes of the first, second and third order all contained casts. The lung contained air mixed with mucus and pus. The trachea contained casts which terminated on the under surface of the epiglottis. The physician questioned whether it was an ascending bronchitis or a descending fibrinous exudation from the pharynx downwards, because in this case, as in his own, there had been tonsilitis about a week before the alarming symptoms set in, but arrives at the conclusion that the disease was primary in the bronchial tubes. He had thus been fortunate enough to observe this rare disease in two cases, one representing the chronic, the other the acute form, and both of them conforming to the rule laid down by Doctor Davis, that the acute generally terminates fatally, while the chronic in many cases terminates favorably. Both of his cases were of the male sex, and both were rather given to alcoholic stimulants.

PROFESSOR E. FLETCHER INGALS said he had had very little experience in these cases. It has never been his bad fortune to meet with a case of acute diphtheritic bronchitis, except those growing out of ordinary diphtheria, which are unfortunately frequent. He had treated three cases of the chronic form of the disease, none of them very severe. In each case there was from time to time expectoration of the croupous deposit, but he did not see them at any time when they were very ill. The history of the chronic cases is that they will have acute attacks from time to time for months or years, running from ten days to two weeks. They almost universally recover from these, though occasionally they die of phthisis. The acute cases, as a rule, die, though from 25 to 50 per centum are said to recover. A friend in the country sent him last winter casts from a large number of the bronchial tubes in which there must have been five or six branches to the casts. They had been coughed up by a patient of his who subsequently recovered.

DR. C. M. FITCH said some eight years ago he had a case of this kind in a lady about to be confined. When he first saw her she was in an almost comatose condition, the face livid, and she was almost pulseless, the respiration fearfully obstructed, although air was entering all the larger bronchi. The woman died a few hours later, the child being born after she had become entirely unconscious. Four or five days later a child of the same family was taken with diphtheritic croup, the membrane passing down through the larynx, when Doctor Fenger performed the operation of tracheotomy. He had no doubt that the lady's case was one of acute croupous bronchitis.

PROFESSOR JOHNSON, in closing the discussion, said, in reply to Professor Babcock as to the influence of the potassium and mercurial salts upon the exudate, not only was the expectoration frothy, but after using these medicines for some time the casts became thin and evidently diminished in amount before they were thrown off. It was his opinion that the mercurial salts and the potassium had some influence in diminishing the amount of the exudate. It be will remembered that from some time in December until February there were no casts thrown off. Some time before that the patient had been taking potassium and mercurial salts. In estimating the frequency of this disease, he had excluded cases which seemed to be of diphtheritic origin; he had seen several cases where there had been well-marked casts thrown off; in one case there were seven successive discharges from the bronchial ramifications of one lung. Those were diphtheritic and were from a child who was recovering from bronchial diphtheria. He does

[1] Vol. iv, fasc. 4, 1872.

not say that this is not in its character anything like diphtheria, but he thinks there is a radical difference between the membrane and that which forms in diphtheria. It does not seem to him that we have the same tendency of disintegration that we have in diphtheritic forms of exudation; the exudate is more plastic. He had purposely not discussed the acute form, as he had not had an opportunity to study such cases. The notes here recorded were made in the presence of the patient, and they seemed to him worthy to be put on record.

PROFESSOR H. A. JOHNSON read a paper on

PNEUMATIC DIFFERENTIATION AND MEDICATION, which appeared in the January number of the *Journal and Examiner*.

PROFESSOR E. FLETCHER INGALS asked Professor Johnson if he thought the patient would get more of the medicated vapor into the lungs with the compressed air than with ordinary air.

PROFESSOR H. ROBERT BABCOCK said it has always been his opinion that pneumatic differentiation is essentially the same as the administration of compressed air, and he had not found reason to change his opinion. However, in justice to the inventor, he would like to ask Professor Johnson what he thinks of Mr. Ketchum's assertion that the rarefaction of the air around the chest of the patient by lessening atmospheric pressure allows the chest, and therefore the residual air, to expand, and with this expansion of the residual air lessens the resistance to the tidal air. In other words, that if the residual air did not expand, the tidal air would meet with resistance from the residual air as from an air:cushion; also that this expansion of the residual air tends to force out any little plugs of mucus which may have obstructed the bronchi; that in this respect pneumatic differentiation certainly accomplishes more than could be done by the inhalation of compressed air.

PROFESSOR JOHNSON said the claim of a special value in the cabinet as a means of differentiation is based upon a fallacy, viz., the assumption of a *vis a fronte*. If you take the pressure off from the outside of the chest there is a kind of force that drives the fluids to the surface of the body. Suppose you reverse the case, place the patient outside and let him breathe the rarefied air, is there a *vis a fronte?* It certainly seemed to him that there is no such thing. It is a *vis a tergo* that pushes air into the lungs, and a *vis a tergo* that pushes the air out of the lungs, and the equilibrium is maintained. As to the effect of the rarefied air in the cabinet upon the residual air, it is about the same as going up and down in an elevator of one of our tall buildings. In answer to Doctor Ingals it would seem to him that more spray may be carried into the bronchial tubes in the stream of condensed air than in air inhaled without differentiation of pressure. The discussion is simply upon physiology and physics, not the value of pneumatic differentiation. Mr. Ketchum or some one connected with him, invented that term, but the thing itself, taking the name away, is by no means new.

DR. FRANKLIN H. MARTIN read a paper on

ELECTROLYSIS IN THE TREATMENT OF FIBROID TUMORS OF THE UTERUS, with a description of Doctor Apostoli's methods.

He considered his subject under the following heads:

1. Consideration of the tumor.
2. Means of generating a current.
3. Electrodes, connections and other apparatus.
4. Electrolytic action of the current.
5. Cataphoric action of the continuous current.
6. The difference in the local action of the two poles with powerful currents.

7. Operation and details of application, with a description of Doctor Apostoli's methods.

He divided fibroid tumors of the uterus according to their position, into submucous, interstitial and subperitoneal, according to their condition into hæmorrhagic and non-hæmorrhagic. Any means of generating a continuous uninterrupted current of electricity of 200 milliampere strength that is practicable will answer all the requirements for electrolytic treatment of fibroid tumors of the uterus. Professor Martin uses a battery composed of 115 crow-foot gravity cells for this purpose, from which he can easily get a current of three or four hundred milliampére strength when properly charged. Storage cells and the dynamo were mentioned as possessing a future for this kind of work.

The very strong current that one now used in operations of this kind is made practicable by improvements in electrodes and conduction. Doctor Apostoli overcame the pain caused by high tension currents by using as a surface electrode a thick paste of potter's clay spread upon the abdomen with proper connections from a plate of soft metal upon its free surface. This answers the purpose perfectly, but has the objection of being very troublesome and very inelegant. Professor Martin presented a decided innovation in this direction in the way of a surface electrode. From a soft plate of metal, the margins of which are bent so as to form a concavity upon one surface of an inch in depth, he has constructed an electrode by stretching loosely over this concavity an animal membrane, making the surface between the membrane and the metal water-tight. Through a stopper in the metal surface the inter-surface is filled with a warm saturated solution of chloride of sodium. This contrivance with its membranous surface upon the abdomen, with proper connections from the metal surface, possesses all the advantages of the potter's clay electrode of Dr. Apostoli, with none of its disagreeable features. The author has been able to use a current of 150 milliampére strength by means of the above electrode without producing the slightest discomfort. The internal electrodes include a uterine sound of platinum with the intra-vaginal portion insulated, a sharp probe of platinum and iridium with insulating sheath, and a number of needles insulated with hard rubber to within an inch of the point.

The electrolytic action of the current was dwelt upon at length and given its due prominence.

The cataphoric action of the galvanic current was described, and to its action the author ascribed considerable prominence as aiding absorption of the fibroid growths.

*Galvano-caustique* is the name given to the local effect of the two poles when used intra-uterine in the form of metal probes. This effect is considered a point of great importance by Doctor Apostoli, and from which he expects to see a great advance developed in the treatment of the hæmorrhagic fibroid tumors of the uterus. The effect referred to is only obtained from a current of 100 milliampére strength concentrated by means of an electrode of unattackable metal. The local effect of the two poles upon the lining of the uterus are distinctly different. The effect of the positive pole is to coagulate and harden the tumor that it comes in contact with without changing the vitality of the tissue. The effect of the negative pole is to cause a liquefaction of the tissues in which it comes in contact, with considerable destruction of tissue.

The coagulating effect of the positive pole is utilized in transforming the hæmorrhagic surface of the endometrium into a dry, coagulated surface that will not allow of a hœmorrhagic exudate.

Three operations or methods of application of these powerful doses were described in detail.

1. Intra-uterine *galvano-caustic.*
2. Negative cervical galvano-puncture.
3. Galvano-puncture or needle-operation: (a) extra-peritoneal; (b) intra-peritoneal.

The first operation (intra-uterine galvano-caustic) is employed for two effects: 1st, to check excessive hæmorrhage by the local effect of the positive pole upon the endometrium; 2d, for the reduction in size of the tumor by the electrolytic action of the powerful current as it passes through the tissue of the tumor.

The second operation (negative cervical galvano-puncture) is performed for the purpose of establishing an artificial channel into the substance of the growth, to take the place of the cervical canal that has been distorted or obstructed to such an extent that it cannot be entered for the ordinary intra-uterine treatment. The effect sought here is the reduction of the tumor.

The third operation described by the author of the paper was the galvano-puncture, or needle-operation proper. This is for the treatment of large sub-peritoneal fibroids that can only be reached by means of needles, and the electrolytic action only of the current is expected.

The author offered the following summary of conclusions :

1. A means of generating a continuous current of electricity which can be increased from 10 to 250 milliampere strength is necessary in order to obtain all the effects of the electrolytic treatment of fibroid tumors of the uterus.

2. The most distressing hæmorrhages from fibroid tumors can be healed by the local coagulating effect of the positive pole if applied intra-uterine.

3. The intra-uterine electrode, when positive, should be of unattackable metal and should conform, as nearly as possible, to the size and shape of the uterine canal, and have the intra-vaginal portion insulated.

4. When the cervical canal cannot be entered, a negative galvano-puncture should be made into the presenting part of the obstructing mass of the tumor, and an artificial channel created which is to take the place of the impenetrable uterine canal in all subsequent treatments.

5. The intra-uterine electrode should in all cases be negative, unless there is hæmorrhagia or excessive leucorrhœa, when the positive pole is required.

6. The strength of the current should be the strongest possible consistent with the desired therapeutic effect, and the toleration of the patient.

7. Cases of intolerance of high doses arrange themselves under the three following heads: 1, acute hysteria; 2, acute enteritis; 3, acute metritis, peri- or pari-metritis. The most tolerant are the deep uteri and profusely hæmorrhagic.

8. The ordinary duration of the séance should be about eight minutes.

9. The number of operations are necessarily dependent upon and influenced by the result to be accomplished. A severe hæmorrhagia can be checked in from four to five treatments, while a general reduction of the tumor necessitates many, varied, of course, according to the size and location of tumor. In many cases simply a restoration to health and a relief from the prominent and annoying symptoms must be accepted as the substitute for an actual cure.

10. The time of commencing treatment matters little if the tumor is not rapidly growing and no excessive hæmorrhage is present. The operation should be intra-menstrual, if possible, but if hæmorrhage is continuous, operate during flow.

11. Extra-uterine puncture should be regarded only as a last resort; but every means of reaching the tumor through the uterus being impracticable, seek, if possible, to make the operation extra-peritoneal ; should this, in turn, prove equally undesirable, use as a final alternative the abdominal puncture.

12. Strict antiseptic precautions should be carried out in this treatment, as in all others.

DR. H. T. BYFORD said he wished to express his admiration of the mastery which Doctor Martin has acquired over this method of treatment. It will undoubtedly take an important place among the surgical remedies for uterine fibroids. But the usefulness of any remedy must be determined not only by its effect, but also by its safety and convenience of employment. For instance, the radical treatment or removal of the tumor, is a frightfully severe measure, and is reserved for cases that cannot be cured in any other way, while removal of the appendages is attended with considerable risk of life, and is reserved for those cases which cannot be helped by less dangerous means. So also this electrolysis, which is difficult of application and capable of harm, must be reserved for cases which cannot be cured by remedies more convenient and less hazardous. But before this, the safest of surgical procedures, can become the usual treatment for such cases, it must prove more efficacious than the still safer medicinal treatment. Fibroid tumors with hæmorrhage are the ones also most benefited by ergot and tampons in the majority of cases, and after the hæmorrhage is thus relieved there is no hurry in resorting to surgery. As to the effect of this treatment on the tumor itself, the evidence is not all in. The success of Apostoli, whose persistence is very praiseworthy, has been somewhat limited with regard to the removal of the tumor. The success of the so-called electrolysis upon fibroids seems to be no better, if as good as that of ergot, and it is doubtful if it is produced in a very much different way. That electrolysis can break up the chemical constituents of a living tumor for any considerable distance from the electrode, without destroying the life and absorbing properties of the tissue in the course of the current, is a theorem that requires more than mere mathematical demonstration. We all believe, however, that it can stimulate the uterus to a contraction and partial strangulation of the tumor, and also stimulate the absorbents to remove it, and are prepared to have it proven to us that it will do so in many cases in which ergot fails. But it happens that the cases in which ergot is of the least use are those in which electrolysis is of most difficult application—the subperitoneal. And yet he has, as a rule, relieved the symptoms of the subperitoneal variety (excepting those immense neglected ones too large for anything but removal) by ergot, which, even when it cannot cause compression of the tumor, can diminish its blood supply. He hopes and believes, however, that the methods of Apostoli will enable us to manage safely cases that must otherwise require a dangerons operation.

PROFESSOR WILLIAM T. BELFIELD said: Doctor Martin has conferred a favor upon us in bringing Apostoli's method before us. During the last twenty-five years various attempts have been made to reduce fibroids of the uterus by the galvanic current; yet none of them have been recognized as successful, because, doubtless, as Doctor Martin very properly says, the current has been used in an ignorant, inaccurate and bungling way. Apostoli's method is unique in the strength of the current used, as well as in the accuracy of its application to the uterus; whether this method will produce satisfactory results remains, as it seems, to be seen, for Apostoli himself has been guilty of the gross inaccuracy which necessarily impairs the confidence claimed by his statements. Through Doctor Martin's kindness he had an opportunity to read Apostoli's book in which are set forth his methods and the results of treatment on ninety-eight patients. The book bears the impress of perfect candor and truth, and yet, in looking through the ninety-eight cases which he records as fibroids of the uterus, most of which were benefited, it is evident that nearly all were not fibroids, but were cases of subinvolution. Many of them were women under 30 years of age belonging to the poorer classes, and who were unable to secure proper rest and care after confinement. The clinical history as well as the physical examination recorded by Apostoli shows that they were plain cases of subinvolution. That the electric current has a value in such cases is unquestionable and has often been demonstrated, but it is not accurate nor justifiable to apply the results obtained in treating subinvolution to the treatment of fibroids, for the distinction between subinvolution and myoma is as sharp anatomilically and etiologically as it is clinically. It seemed to him that only four of Apostoli's cases were evidently and certainly uterine fibroids; it is possible that six others might be so reckoned, but certainly eighty-eight of the cases were, so far as can be judged from the record, cases of mere subinvolution. Yet the results secured in these four cases were most positive and decisive. In two of them the tumors were of enormous size. These two patients had been seen by Pean, whose opinion was sought as to the advisability of operation. In one case he declined to operate, on the ground that the patient could not survive an operation; in the other he was ready to operate, but the patient declined. Yet both of these cases of undoubted and extensive myomata were rapidly and greatly improved and the tumors reduced under Apostoli's treatment. But four cases are not enough to justify a generalization, and we may hope that Doctor Martin will soon favor the Society with the results of the method in his own hands, for we may be sure that he will avoid the sole error which can be charged against Apostoli.

PROFESSOR P. S. HAYES said he had not given this matter a trial in fibroid tumors, but had in the use of electricity generally. There are two factors in the treatment of fibroids by electrolysis—the chemical and physical and the physiological. How the physiological action of electricity operates is a question, but the chemical and physical produce their effect by the splitting up of compound molecules and the chemical and physical liberation of gases at the polls. Certainly this factor (the chemical and physical) can be measured by the amount of chemical work done, which depends upon the strength of the battery, etc. He said it seemed to him that a current even of 50 milliampères is rather strong to use in the beginning. He had had quite an extensive experience, and could call to mind six or eight cases in which even the slightest galvanic current would produce dizziness so that the patient would have to lie down for half an hour. In one case in which only a half a dozen of cells were used, the patient left his office staggering like a drunken woman, and finally had to go in some place and rest. Afterwards he was unable to treat her except by allowing the current to traverse a very minute portion of her body. An induction apparatus the helix of which consists of thick wires and in which the current is generated by a battery of very large surface, gives a current which resembles somewhat the interrupted galvanic current; it stands midway between the ordinary induced current and the interrupted galvanic current of eight or ten cells. This current, applied over a tumor sufficiently strong to produce vigorous contractions, will reduce the size of a fibroid when ergot has failed to do so, and apparently in the same way, by inducing forcible uterine contractions and contractions of the abdominal muscles, tending to astringe it and squeeze out the life-blood which enters into it. It seemed to him, from his experience, that the injection of a few minims of 95 per centum carbolic acid into the tumor would produce a very similar local result to that produced by either the positive or negative pole. In the *Medical Record*, of recent date, he had seen an abstract of the last paper of Apostoli in which it states that, while he has reduced fibroid tumors in size, relieved the distressing symptoms and made the patient very much better, yet he does not claim that he has ever absolutely removed a fibroid so that it could not be detected. This method of treatment seems to offer a good deal of hope in many cases where other means have failed, and he would not hesitate to try it himself and recommend others to do so.

PROFESSOR H. MARTIN, in closing the discussion, said he could not agree with Doctor Byford that this method of treating fibroid tumors should be reserved as a last resort. In careful hands it is entirely free from danger, pain, and (except in the last variety of operation described this evening) all disagreeable features, and it should not, therefore, be postponed until less efficient and more objectionable means have been employed, such as the hypodermic injection of ergot, its administration internally in large quantities, tamponing the vagina, cauterization of the uterine mucous membrane, and innumerable other less efficient means of relief. In his experience with the strong current he had never yet seen an untoward result, and he had employed currents varying in strength from 25 to 250 milliampéres. Apostoli's method should especially be adopted early for the checking of hæmorrhage from the uterine cavity. He could not agree entirely with Doctor Belfield's conclusion in regard to Doctor Apostoli's results as reported in 1884.[1]

[1] That Doctor Belfield is in error in regard to this point will be made very apparent by the following summary of the cases reported by Doctor Apostoli and referred to by Doctor Belfield; 94 cases were reported; 15 of the cases had never borne children. Of the remaining 79 cases, where subinvolution of the uterus was possible, we find 4 pedunculated subperitoneal tumors in which error of diagnosis was not probable. Of the remaining 75 cases, 9 measured from 10 to 21 cm., and the accompanying descriptions of the cases can leave no doubt in an unprejudiced mind in regard to their being large fibroid growths. The full description in detail of the remaining 66 will convince anyone who will take pains to peruse them, that at least 44 are well defined fibroids. This leaves but 22 cases of the 94 reported in which there are not distinctive characteristics described, which definitely distinguishes them from simple subinvolution of the uterus.

His own results had been such as to make him very sanguine in regard to the value of strong currents for the relief of these difficulties, but as the object of the paper is the discussion of the method, and not the results, their consideration would be postponed until another time. He had never seen troublesome dizziness occur in the use of these currents in treatment about the abdomen.

